# Polymerization‐Induced Self‐Assembly for the Synthesis of Polyisoprene‐Polystyrene Block and Random Copolymers: Towards High Molecular Weight and Conversion

**DOI:** 10.1002/marc.202400727

**Published:** 2024-10-26

**Authors:** Maryam Moradi, Prokopios Georgopanos

**Affiliations:** ^1^ Helmholtz‐Zentrum Hereon Institute of Membrane Research Max‐Planck‐Straße 1 21502 Geesthacht Germany

**Keywords:** emulsion polymerization, PISA, polyisoprene‐polystyrene copolymers, RAFT

## Abstract

In this study, reversible addition‐fragmentation chain‐ transfer (RAFT) polymerization combined with the polymerization‐induced self‐assembly (PISA) technique is used to synthesize polyisoprene (PI)‐based block and random copolymers with polystyrene (PS), aiming for high molecular weight and monomer conversion. The focus is to optimize the polymerization conditions to overcome the existing challenge of cross‐linking and Diels‐Alder reactions during the polymerization of isoprene, which typically constrain the reaction conversion and molecular weight of the final polymers. Using a poly(methacrylic acid) (PMAA) macroRAFT agent synthesized in ethanol at 80 °C, random and block copolymers of PS‐PI with a target molecular weight of 50 000 g mole^−1^ and a high monomer conversion of ≈80% are achieved under optimized conditions in water‐emulsion at 35 °C. ^1^H nuclear magnetic resonance (NMR) verified the successful synthesis as well as the high content of *1,4* microstructure in polyisoprene. The thermal analysis via differential scanning calorimetry indicated distinct glass transitions for the microphase‐separated PI‐PS block copolymer, while a single transition for PI‐PS random copolymer, indicating no microphase separation. Furthermore, dynamic light scattering analysis together with transmission electron microscopy provided further insight into the self‐assembled emulsion nanoparticles of the polymers indicating a particle size in the range 70 to 130 nm.

## Graphical abstract

This study explores the synthesis of polyisoprene (PI) homopolymers, polyisoprene‐polystyrene (PI‐PS) block and random copolymers via RAFT‐PISA polymerization in water. Using a poly(methacrylic acid) macroRAFT agent, PI and PI‐PS copolymers with high monomer conversion are achieved. Thorough characterization indicated the successful synthesis, emphasizing PISA's scalability for producing tailored PI‐PS copolymers.

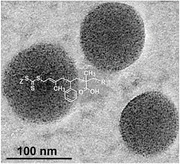

## 1 Introduction

Copolymers of polyisoprene (PI) and polystyrene (PS) are an attractive class of materials that provide a broad spectrum of mechanical, thermal, and chemical capabilities by combining the intrinsic properties of the two blocks. The resulting materials’ composition and structure can be well‐controlled throughout the copolymerization process, allowing for the customization of their features to suit a particular target. While polystyrene offers rigidity, transparency, and thermal stability, polyisoprene adds elasticity, robustness, and flexibility at low temperatures, making the copolymers suitable for packaging, construction, automotive, and biomedical industries.^[^ ^1^, ^2^, ^3^, ^4^, ^5^ ^]^

Anionic polymerization is well‐known for producing well‐defined PS‐*b*‐PI block copolymers with precise control over molecular weight and molar mass distribution.^[^ ^3^, ^6^, ^7^, ^8^, ^9^, ^10^, ^11^ ^]^ However, its adoption is limited by the need for specialized equipment and its high sensitivity to moisture and electrophilic impurities.^[^ ^7^, ^12^ ^]^ Alternatively, controlled radical polymerization including nitroxide‐mediated polymerization (NMP),^[^ ^4^, ^13^ ^]^ and RAFT polymerization^[^ ^14^, ^15^ ^]^ can be exploited for developing PI block copolymers. NMP, however, requires high temperatures, which increases the risk of cross‐linking and loss of control over molecular weight dispersity.^[^ ^16^ ^]^ In contrast, RAFT polymerization can be conducted at lower temperatures, offering a more controlled alternative.^[^ ^17^ ^]^ It should be noted that the synthesis of PI homo‐ or copolymers with PS using controlled radical polymerization requires tackling the challenges of the very low polymerization rates of resonance‐stabilized styrenic and allylic monomers, such as styrene and isoprene (propagation rates *k*_p_^styrene^ > 240 *k*_p_^isoprene^ < 100 L M^−1^ s^−1^ at 50 °C).^[^ ^1^, ^18^ ^]^ More importantly, the Diels‐Alder dimerization of isoprene monomers and crosslinking reactions of the diene‐bond in PI, notably at high molecular weights and monomer conversions should be controlled during the polymerization.^[^ ^1^, ^17^, ^19^, ^20^, ^21^ ^]^ These obstacles lead to polymers with low conversion or low molecular weight. For example, Langer et al. used bulk RAFT polymerization at 110 °C to synthesize P(S‐*co*‐I) with 20% isoprene and could achieve 39% conversion after 24 h (apparent molecular weight measured by gel permeation chromatography (GPC)*, M̅*_n_^GPC^ = 49 000 g mol^−1^, *Ð* = 1.32).^[^ ^22^ ^]^ Gramlich et al.^[^ ^23^ ^]^ carried out RAFT bulk polymerization of isoprene with hydroxy functional monomers at 125 °C for 24 h, which resulted in 45% to 50% conversion and an apparent *M̅*_n_^GPC^ between 6100 and 6600 g mol^−1^ and *Ð* values of 1.3 to 1.4. They noted 25% isoprene consumption due to Diels‐Alder homodimerization. In another study, bulk RAFT polymerization of isoprene at 125 °C yielded PI with 27% conversion, *M̅*_n_^NMR^ of 4670 g mol^−1^, *M̅*_n_^GPC^ of 9200 g mol^−1^ and *Ð* value of 1.25 after 25 h.^[^ ^12^ ^]^ Additionally, using PEO macroRAFT for chain extension with PI, copolymers (38% to 71% PI) were prepared with a *M̅*_n_^GPC^ ranging from 5500 to 18 000 g mol^−1^, *Ð* = 1.28 to 1.34, and ≈30% conversion after 24 h at 125 °C.^[^ ^24^ ^]^ Lauterbach et al.^[^ ^25^ ^]^ conducted ultrafast isoprene photo‐iniferter RAFT polymerization at 145 °C under UV, achieving 80% conversion in 100 min, *M̅*_n_^NMR^ of 5900 g mol^−1^, and *Ð* = 1.5. They also prepared P(S‐*r*‐I) copolymer (*M̅*_n_^GPC^ = 6600 g mol^−1^, *Ð* = 1.4), PS‐*b*‐PI copolymer (*M̅*_n_^GPC^ = 17 300 g mol^−1^, *Ð* = 1.4), and PS‐*b*‐PI‐*b*‐PS (*M̅*_n_^GPC^ = 25 400 g mol^−1^, *Ð* = 1.5)

Surfactant‐free RAFT emulsion polymerization, also known as polymerization‐induced self‐assembly (PISA), is an environmentally friendly and scalable technique. It reduces reliance on organic solvents, improves heat transfer, and enhances process safety while allowing the synthesis of polymers with various block units.^[^ ^26^, ^27^, ^28^, ^29^, ^30^, ^31^, ^32^ ^]^ More importantly, compartmentalization within micelles significantly enhances the reaction rate and conversion compared to non‐compartmentalized methods, e.g., solution or bulk polymerization.^[^ ^26^, ^33^, ^34^ ^]^ Additionally, this technique eliminates the need for surfactants, which can be toxic and require removal from the final product.^[^ ^28^, ^35^ ^]^ To synthesize block copolymers using the PISA technique, a hydrophilic polymer block (macro‐RAFT agent) is first prepared through RAFT solution or bulk polymerization. Then, a hydrophobic monomer is polymerized via RAFT emulsion/dispersion polymerization using the prepared hydrophilic macro‐RAFT agent. The resulting amphiphilic copolymers self‐assemble into micellar structures in water.^[^ ^35^, ^36^, ^37^ ^]^ Despite its advantages, surfactant‐free RAFT emulsion polymerization of isoprene and its copolymers remains relatively underexplored, with few studies dedicated to this area. Bar‐Nes et al.^[^ ^16^ ^]^ synthesized triblock copolymers of poly(acrylic acid‐*co*‐styrene‐*co*‐isoprene) (PAA‐*co*‐S‐*co*‐I) using the PISA mechanism. They achieved a 75% conversion for PAA_10_‐S_45_‐I_80_ after 2 h at 60 °C. The theoretical molecular weight based on the constituent units is ≈10 900 g mol^−1^. However, in order to attain desirable mechanical properties, higher molecular weights are necessary.

This study explores the synthesis of poly(I‐*co*‐S) random and block copolymers using PISA polymerization technique. The primary objective is to investigate and fine‐tune the polymerization conditions, to minimize/avoid cross‐linking reactions and to achieve copolymers with high molecular weights (50 000 g mol^−1^) and high monomer conversion compared to previous studies. Additionally, we thoroughly characterize the emulsion and copolymer composition, as well as to investigate the thermal properties of the synthesized polymers. This research significantly contributes to straightforward, practical, efficient and more environmentally friendly synthesis of P(I‐*co*‐S) copolymers.

## 2 Results and Discussion

### 2.1 Synthesis of Polyisoprene via Pisa and Polymerization Optimization

**Figure 1:**
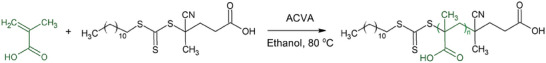
Synthesis path of PMAA macroRAFT using solution polymerization.

Several hydrophilic macroRAFT agents such as poly(ethylene oxide) (PEO),^[^ ^40^ ^]^ poly(3‐vinylpyridine) (P3VP),^[^ ^28^ ^]^ poly(acrylic acid) (PAA)^[^ ^41^ ^]^ and poly(methacrylic acid)^[^ ^42^ ^]^ has been used for PISA polymerization, among which PAA has been shown as a potential macroRAFT agent for the polymerization of styrene and isoprene through PISA emulsion polymerization.^[^ ^16^ ^]^ In our study, a PMAA‐based macroRAFT agent was prepared owing to the lower mid‐chain radical effect (due to hydrogen‐atom abstraction) compared to PAA, which can reduce the polymerization rate.^[^ ^16^, ^43^, ^44^, ^45^ ^]^ Furthermore, 4‐cyano‐4‐[(dodecylsulfanylthiocarbonyl)sulfanyl]pentanoic acid (CDTPA) has been selected as an efficient RAFT agent in the polymerization of isoprene owing to its high transfer coefficient (C_tr_), which refers to the frequency of exchanges between the propagating species and species containing RAFT agent.^[^ ^17^ ^]^ Indeed, CDTPA with higher C_tr_ tends to provide a uniform growth of the propagating chains.^[^ ^17^ ^]^ **Figure** **1** and Figure S1 (Supporting Information) display the synthesis path and ^1^H NMR spectrum of the macroRAFT agent consisting of PMAA and CDTPA, respectively.

**Figure 2:**
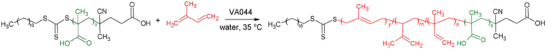
Synthesis path of PMAA‐b‐PI diblock copolymer using PMAA macroRAFT agent via surfactant‐free PISA‐RAFT emulsion polymerization.

Then, the synthesized PMAA macroRAFT was initially used to polymerize isoprene to determine the optimal polymerization conditions. **Table** **1** represents various examined polymerization conditions. The initial trial of polymerization (sample 1) resulted in a very high polymerization conversion of ≈93%, however, the final dried product was insoluble in (tetrahydrofuran) THF. The solubility challenge of PI synthesized via PISA was earlier reported by Bar‐Nes *et. al*.^[^ ^16^ ^]^ This could originate from crosslinking within the PI backbone during the radical polymerization.^[^ ^46^ ^]^ In fact, the remaining double bonds in PI backbone are thermally unstable, rendering them susceptible to cleavage and subsequent reactions with both neighboring double bonds, propagating radical species and/or initiator.^[^ ^46^, ^47^ ^]^ Hence, our initial focus was on optimizing the polymerization conditions to reduce/avoid crosslinking and overcome the solubility issue. The reduction of radicals’ concentration, i.e., initiator to macroRAFT ratio in the subsequent polymerizations (samples 2 and 4), led to an enhanced, yet not complete, solubility of the final dried PI in THF. Therefore, the initiator to macroRAFT ratio was adjusted to 1/10 for further formulations. Next, to reduce the possibility of double‐bond cleavage, the polymerization at a lower temperature was investigated. To this aim, samples 5 to 8 were synthesized using VA044 (2,2′‐azobis[2‐(2‐imidazolin‐2‐yl)propane] dihydrochloride) initiator owing to its lower decomposition temperature (43 °C) compared to ACVA (4,4′‐azobis(4‐cyanovaleric acid) initiator (decomposition temperature 75 °C), which was used for previous samples. Notably, the polymerization at 45 °C yielded PMAA‐*b*‐PI with complete solubility in THF. Nevertheless, due to the lower reaction temperature and, consequently, reduction in the polymerization rate of isoprene compared to the analogous synthesis conducted at 70 °C, a lower conversion was obtained in sample 5 (83% vs. 54%). A prolonged polymerization time of 41 h increased the conversion to 84% (sample 6). However, the longer polymerization time and heat exposure gave rise to crosslinking and insoluble polymer in THF. Then, a further decrease in the reaction temperature to 35 °C was also investigated (sample 7). Notably, at this temperature, below the decomposition temperature of VA044, the concentration of VA044 radicals would be lower compared to polymerization at 45 °C. Therefore, to adjust the radicals’ concentration, we used a higher initiator to macroRAFT ratio of 1/5. This ratio was calculated based on the decomposition rate of the VA044 initiator at 45 and 35 °C, ensuring equivalent concentrations of initiator radicals after 41 h of reaction time, either at 45 °C with an initiator to macroRAFT ratio of 1/10 or at 35 °C with a ratio of 1/5. The plot and calculation parameters can be found in Figure S3 (Supporting Information). The polymerization yielded a high conversion of 70% after 43 h, with excellent solubility of the dried polymer in THF. Afterward, following the same polymerization condition and a comparable adjustment of initiator concentration, the new polymerization (sample 8) was conducted for 48 h to enhance the conversion (The plot and calculation parameters can be found in Figure S4, Supporting Information). Eventually, at a longer polymerization time (53 h), a higher conversion of 82%, along with a complete solubility in THF could be achieved, which was considered as the optimal polymerization condition for PI. **Figure** **2** displays the polymerization reaction of PMAA‐*b*‐PI. Upon analyzing the peak integrals in the ^1^H NMR spectrum (Figure S5, Supporting Information), a microstructure including 89.0% *1,4*, 5.5% *1,2* and 5.5% *3,4* was found for PI (calculation in Supporting Information).

**Table 1:** PMAA‐*b*‐PI with PMAA macroRAFT and target molecular weight of 50 000 g mol^−1^.

Samples	Initiator	[MacroRAFT] /[I]	Reaction temperature [°C]	Reaction time [h]	Conversion [%]	Solubility in THF	*M̅_n_^GPC^* [g mol^−1^]	*Ð*
1	ACVA	5	70	24	93	–	–	–
2	ACVA	10	70	24	83	–	–	–
3	ACVA	10	70	15	70	–	–	–
4	ACVA	15	70	24	78	–	–	–
5	VA044	10	45	24	54	+	37 600	2.6
6	VA044	10	45	40	84	–	–	–
7	VA044	5	35	43	70	+	65 600	1.8
8	VA044	5.3	35	53	84	+	71 600	1.7

### 2.2 Synthesis of the Ps and Pi Random and Block Copolymers

**Figure 3:**
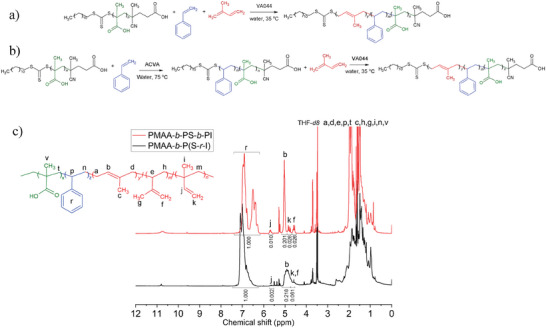
Synthesis path of a) PMAA‐b‐P(S‐r‐I) and b) PMAA‐b‐PS‐b‐PI copolymers via surfactant‐free PISA‐RAFT emulsion polymerization. c) 500 MHz ^1^H NMR spectra of PMAA‐b‐P(S‐r‐I) (black) and PMAA‐b‐PS‐b‐PI (red) in THF‐d8.

Following the optimized polymerization conditions of polyisoprene with high conversion and molecular weight and complete solubility in THF, our efforts directed toward the synthesis of PI and PS random and block copolymers. This expansion of our synthetic endeavors aimed to the versatility and potential applications of PS and PI copolymer architectures. To this aim, first PMAA‐*b*‐P(S‐*r*‐I), copolymer was synthesized, using initiator/PMMA macroRAFT ratio of 1/5 at 35 °C and a target molecular weight of 50 000 g mol^−1^ with a composition of 40% PS and 60% PI. Remarkably, a conversion of 82% was achieved after 48 h together with a complete solubility in THF of the dried product. The polymerization reaction is shown in **Figure** **3**a (for simplicity only *1,4* isomer was drawn) and Figure 3c (black) represents the^1^H NMR spectrum. Notably, the ^1^H NMR spectrum clearly confirms the random structure of S and I in the polymer backbone. First, the aromatic hydrogens of the styrene in the random copolymer appeared as a single broad peak spanning from 6.3 to 7.3 ppm in contrast to PMAA‐*b*‐PS (Figure S8, Supporting Information), where peaks of the ortho hydrogens (6.3 to 6.8 ppm) and meta + para hydrogens (6.8 to 7.3 ppm) revealed separately. It is because ortho‐protons’ peak appears when the styrene block is longer than roughly two to three units. As the randomness of the copolymers increases and styrene sequences get shorter, the ortho‐protons shift toward the low field, where meta + para resonance peaks are located.^[^ ^48^ ^]^ Additionally, compared to ^1^H NMR spectrum of PMAA‐*b*‐PI (Figure S5, Supporting Information) in which methylene peaks of the *1,4*, *1,2* and *3,4* units of the PI are clearly separated, in the random copolymer they have overlapped. This is due to the peak broadening of *1,4* resonance peaks by the immediate neighboring styrene units in the randomized copolymers of PS and PI.^[^ ^48^ ^]^ According to the peak integral analysis in ^1^H NMR spectrum, a microstructure consisting of roughly 88% *1,4* and 12% *1,2* + *3,4* was discovered for PI (calculation in supporting information), which is in good agreement with the microstructure of PMAA‐*b*‐PI. Additionally, the PI and PS content in the PMAA‐*b*‐P(S‐*r*‐I) was roughly estimated by ^1^H NMR spectrum, through dividing the integral of the corresponding styrene to the sum of the corresponding peaks of *1,4* isoprene and styrene (details in supporting information). The calculated value indicated a PS to PI ratio of roughly 45% and 55%, respectively, within the PMAA‐*b*‐P(S‐*r*‐I) copolymer, closely aligned with the initial feed ratio.

Additionally, PMAA‐*b*‐PS‐*b*‐PI with the same ratio of 40% PS and 60% PI and targeted molecular weight of 50,000 g mol^−1^, was intended to be synthesized. The polymerization path is displayed in Figure 3b. This was first started through the copolymerization of styrene with PMAA macroRAFT, followed by chain extension with PI block. This sequential approach in polymerization was chosen to minimize the exposure of the PI block to heating and the possibility of cross‐linking. Therefore, PMAA‐*b*‐PS with a target molecular weight of 23 500 g mol^−1^ was first synthesized through emulsion polymerization reported in the literature,^[^ ^38^ ^]^ yielding a polymer with a full conversion. GPC analysis of the PMAA‐*b*‐PS revealed an apparent *M̅*_n_^GPC^ of 31 000 g mol^−1^ and *Ð* value of 1.5, indicating a good control over polymerization and livingness of the macroRAFT radicals. The synthesized PMAA‐*b*‐PS was then chain extended with PI block, following similar polymerization conditions of PMAA‐*b*‐P(S‐*r*‐I), *i.e*., macroRAFT/initiator ratio of 1/5 at 35 °C. After 24 h the reaction was stopped since visual inspection, such as color and phase homogeneity indicated that a high conversion had been achieved. Notably, the conversion evaluation resulted in a value of 90%, revealing a significantly accelerated reaction compared to PMAA‐*b*‐P(S‐*r*‐I) (80% after 48 h). The increased polymerization rate of isoprene by PMAA‐*b*‐PS macroRAFT compared to PMAA macroRAFT (in PMAA‐*b*‐PI) can be explained by the PISA mechanism. In fact, PISA can be divided into two stages. The first stage involves chain extension of the hydrophilic block with the hydrophobic block to a certain length to become insoluble and lead to micellization. The second stage is the polymerization inside the micelles. It should be noted that the initial chain growth, prior to micellization (stage 1) is slow and depends on the diffusion of hydrophobic monomer molecules from the monomer droplets into the water.^[^ ^37^ ^]^ Considering that, in the case of PMAA‐*b*‐PS macroRAFT agent, micelles are already formed and chain extension occurs via the second stage, which is faster, leading to a higher polymerization rate compared to PMAA macroRAFT agent in PMAA‐*b*‐P(S‐*r*‐I). Thereby, for the copolymerization of PMAA‐*b*‐PI and PMAA‐*b*‐P(I‐*r*‐S) we have used [MacroRAFT]/[I] ratio of 5/1, while for the PMAA‐*b*‐PI‐*b*‐PS we have used [MacroRAFT]/[I] ratio of 10/1 in order to obtain more control over the polymerization^[^ ^49^, ^50^ ^]^ due to the faster reaction kinetic rate.

Eventually, a minor modification of formulation, *i.e*., macroRAFT/initiator ratio of 1/10 (reduced free radical content), led to obtaining PMAA‐*b*‐PS‐*b*‐PI with a reasonably high polymerization conversion of 73% after 24 h, which was soluble in THF after drying. Figure 3c (red) represents the ^1^H NMR spectrum. As illustrated, the ^1^H spectrum of the PS‐PI block copolymer exactly resembles the ^1^H NMR of individual PI (Figure S5, Supporting Information) and PS (Figure S8, Supporting Information) blocks, confirming the block structure. Furthermore, using the ^1^H NMR spectrum the PI and PS content in the PMAA‐*b*‐PS‐*b*‐PI was calculated by dividing the integral of the corresponding hydrogens of styrene to the sum of the integral of the corresponding hydrogen peaks of *1,4* isoprene and styrene (calculation in supporting information). The calculation revealed a PS and PI content of 47% and 53%, respectively, which is in good agreement with the feed ratio. Additionally, a microstructure including 88.0% *1,4*, 6% *1,2* and 6% *3,4* was found for PI, based on the peak integral analysis in the ^1^H NMR spectrum (calculation in supplementary information).

In view of the persistent challenge in copolymerizing isoprene and styrene via radical polymerization, where researchers have traditionally faced an obstacle toward achieving both high molecular weight and high conversion, our developed polymerization protocol represents a significant advancement. This methodology offers a promising avenue to concurrently attain high molecular weight and conversion in isoprene polymerization through PISA technique, which remained previously unattainable to the best of our knowledge.

### 2.3 Thermal Analysis of the Synthesized Materials

The synthesis of PI‐based polymers was well investigated through molecular characterization techniques such as GPC and NMR. However, an essential aspect affecting the application potential of these polymers in various fields, such as coatings, adhesives, or composite materials, necessitates further examination. This aspect involves the thermal and morphological characteristics of the polymers. In this section, we explored the thermal properties of the synthesized polymers through thermogravimetric analysis and differential thermogravimetry (TGA and DTG) and differential scanning calorimetry (DSC) experiments conducted on the PMAA‐*b*‐PI (sample 8, in **Table** **1**), PMAA‐*b*‐PS‐*b*‐PI, and PMAA‐*b*‐P(S‐*r*‐I) samples.

**Figure 4:**
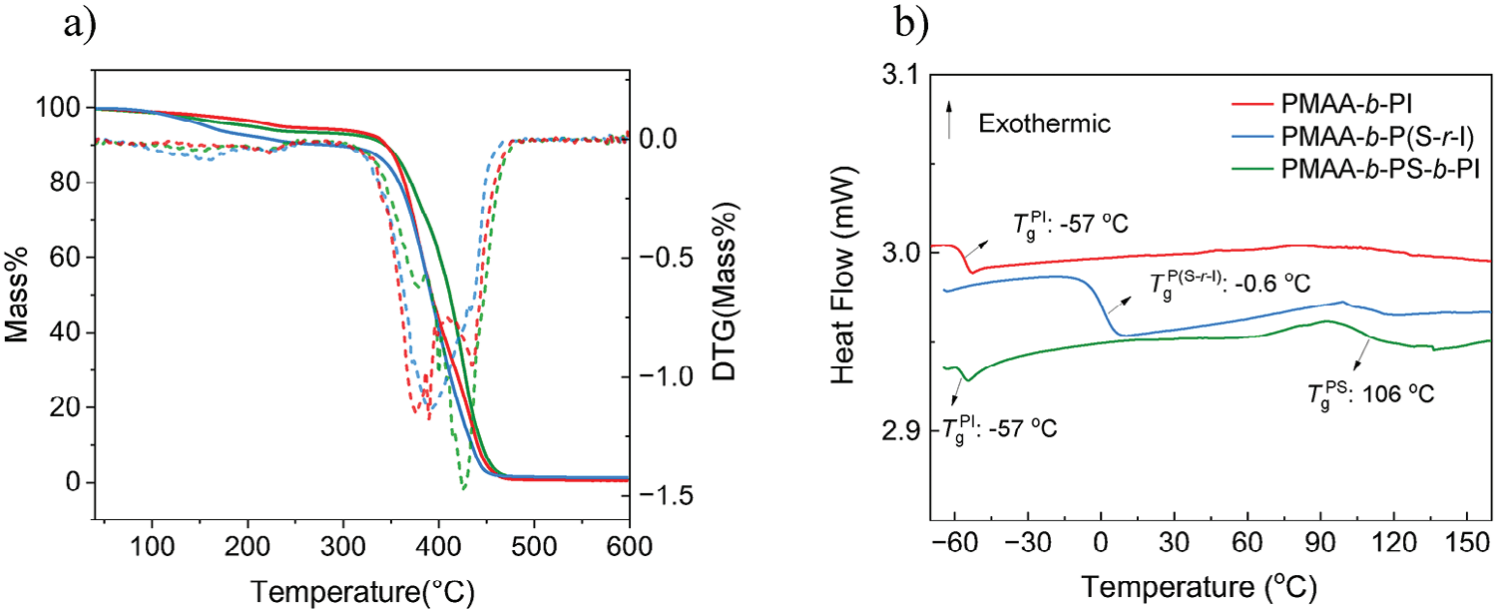
a) TGA analysis of with a 10 °C min^−1^ of (red) PMAA‐*b*‐PI (sample 8), (green) PMAA‐*b*‐PS‐*b*‐PI and (blue) PMAA‐*b*‐P(S‐*r*‐I). The thermograph displays the samples’ relative mass loss as a function of the temperature as well as the derivative of it, providing insights into volatilization, thermal stability and decomposition of the material. In the DTG curves the thermal events are indicated by the minima. b) DSC analysis with a rate of 10 °C min^−1^ under N_2_ atmosphere of (red) PMAA‐*b*‐PI (sample 8), (green) PMAA‐*b*‐PS‐*b*‐PI and (blue) PMAA‐*b*‐P(S‐*r*‐I). The thermograph shows the heat flow as a function of temperature, revealing phase transitions and thermal behavior of the samples.

The TGA curve (**Figure** **4**a) of all samples shows a first thermal event with a mass loss of ≈2% in the range 30 to 120 °C, which is attributed to the evaporation of water bound to the acidic groups of PMAA or residual monomers and solvent.^[^ ^51^ ^]^ The second thermal event, spanning from 120 to 280 °C with a mass loss of ≈6% to 10%, involves mainly the decomposition of unsaturated bonds^[^ ^52^ ^]^ that existed in PI and is in good agreement with linear PI synthesized by another group through anionic polymerization,^[^ ^53^ ^]^ as well as possible further dehydration.^[^ ^51^ ^]^ The third thermal event, occurring at temperatures exceeding 300 °C, corresponds to the decomposition of the remaining bonds. All the three thermal events are verified also by the corresponding minima in the DTG curves.

As is shown in Figure 4b, within a temperature range of ‐70 to 200 °C, no melting or crystallization transitions were observed in the DSC thermographs of the samples. Notably, a prominent thermal phenomenon associated with the glass transition temperature (T*_g_*) could be observed, indicating a transition from a rigid to a rubbery state as temperature increases. The lower T*_g_* appearing at ‐57 °C corresponds to the rigid‐to‐rubbery transition of PI. It is worth noting that the T*_g_* value of PI can significantly vary between ‐100 to 12 °C, depending on the microstructures content in the polymer.^[^ ^54^ ^]^ Based on Widmaier and Meyer's explanation,^[^ ^55^ ^]^ *3,4*‐isomeric microstructure of PI, which features bulky vinyl side branches along with the double bond, contributes to the stiffness of the polymer and restriction in chains movement, shifting T*_g_* values to the higher temperatures. Using Bicerano equation^[^ ^56^ ^]^ the T_g_ of anionically synthesized PI containing *1,4* microstructure with a *M̅*_n_ of 46 000 g mol^−1^ has been calculated to be ‐64 °C.^[^ ^57^ ^]^ Notably, the observed T*_g_* value of our synthesized PI containing 89.0% *1,4*, 5.5% *3,4* and 5.5% *1,2* microstructures at ‐57 °C, fits very well with the predicted value. A slight shift toward higher values in the T*_g_* value is attributed to the presence of *3,4* and *1,2* microstructures within the polymer structure.

Additionally, in the DSC thermograph of PMAA‐*b*‐PS‐*b*‐PI two distinct T*_g_* values clearly appeared. The lower T*_g_* value, appearing at ‐57 °C, is attributed to the elastomeric PI block. On the other hand, the T*_g_* of the hard PS block is observed at 106 °C, signifying the transition from a glassy to a rubbery state, typical of PS. Notably, this distinct separation of T*_g_* values confirms the presence of both PI and PS blocks within the copolymer structure, each exhibiting unique thermal behaviors. In contrast, in the DSC analysis of PMAA‐*b*‐P(S‐*r*‐I), the characteristic T*_g_* transition of PI did not appear, but a new T*_g_* transition emerged at ‐0.6 °C. This observation arises from the strong dependency of T*_g_* on the copolymers’ compositions.^[^ ^10^, ^58^ ^]^ For random copolymers in a bulk state, the T*_g_* is often estimated using the Fox equation and copolymers’ composition as bellow:^[^ ^59^ ^]^

(1) $$\mathrm{FoxEq}.\frac{1}{T_{g}}\frac{w_{1}}{T_{g1}}\frac{w_{2}}{T_{g2}}$$

Here, T_g1_ and T_g2_ represent the glass transition temperatures of the pure homopolymers, while w_1_ and w_2_ denote the respective weight fractions, estimated by ^1^H NMR.

Using the composition of 55% PI and 45% PS in PMAA‐*b*‐P(S‐*r*‐I) and the corresponding T*_g_* of each polymer in the Fox equation resulted in a T*_g_* value of ‐1.8 °C for the copolymer. This result closely aligns with the experimental T*_g_* value of ‐0.6 °C. The minor disparity between the calculated and experimental values may be attributed to differences in the rate of DSC analysis, or chain extension within the polymer. Overall, the DSC analysis conclusively confirms the successful synthesis of both random and block compositions of PS and PI.

### 2.4 Particle Size and Morphology Analysis of the Prepared Elmulsions

**Figure 5:**
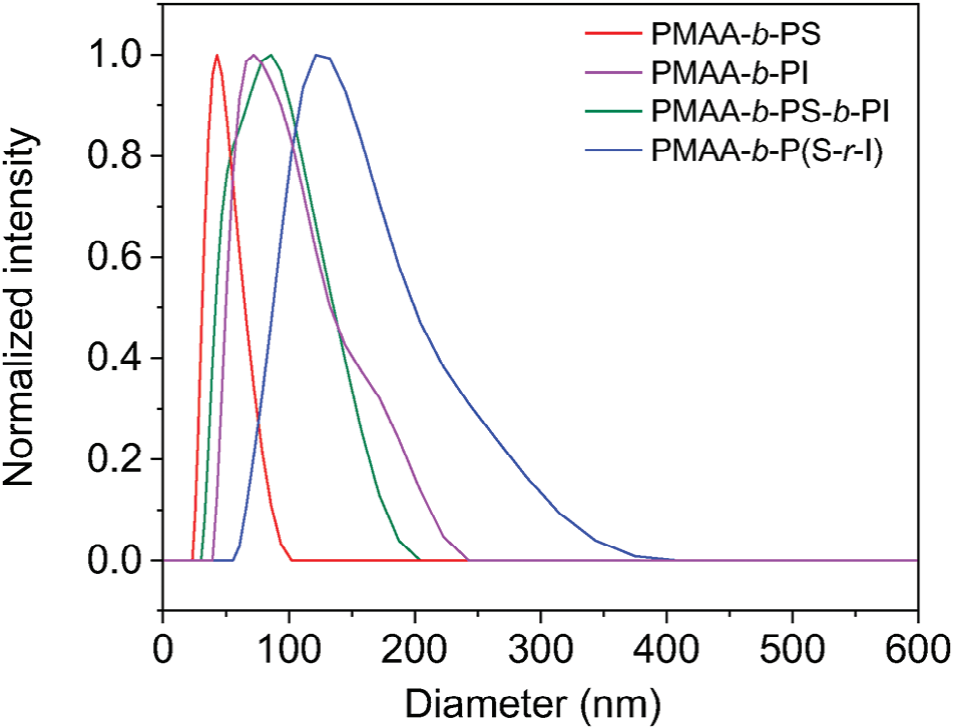
DLS experiment of the PMAA‐*b*‐PI (purple) PMAA‐*b*‐PS (red), PMAA‐*b*‐PS‐*b*‐PI (green) and PMAA‐*b*‐P(S‐*r*‐I) micelles. All the samples were directly taken from the solution emulsion polymerization and diluted to 1% wt.

In order to investigate the impact of the composition and structure of the different synthesized polymers, *e.g*., PMAA‐*b*‐PI, PMAA‐*b*‐PS‐*b*‐PI, and PMAA‐*b*‐P(S‐*r*‐I) on micelles’ morphology of the emulsion, *e.g*., size, shape and distribution, dynamic light scattering (DLS), scanning electron microscopy (SEM) and transmission electron microscopy (TEM) analysis were performed. As demonstrated, in the DLS analysis (**Figure** **5**), PMAA‐*b*‐PI showed a particle size of 72 nm with a roughly double‐sized shoulder, which could be due to the aggregation of particles. PMAA‐*b*‐PS revealed a monomodal size distribution of particles at 43 nm, which increased to 86 nm after chain extension with PI block (PMAA‐*b*‐PS‐*b*‐PI), indicating the success of polymerization. Notably, PMAA‐*b*‐P(S‐*r*‐I) had a larger particle size of ≈132 nm with a broader distribution. This can be explained by the random distribution of S and I in the polymer backbone, rendering any phase separation and self‐arrangement of S and I within the polymer.^[^ ^48^ ^]^

**Figure 6:**
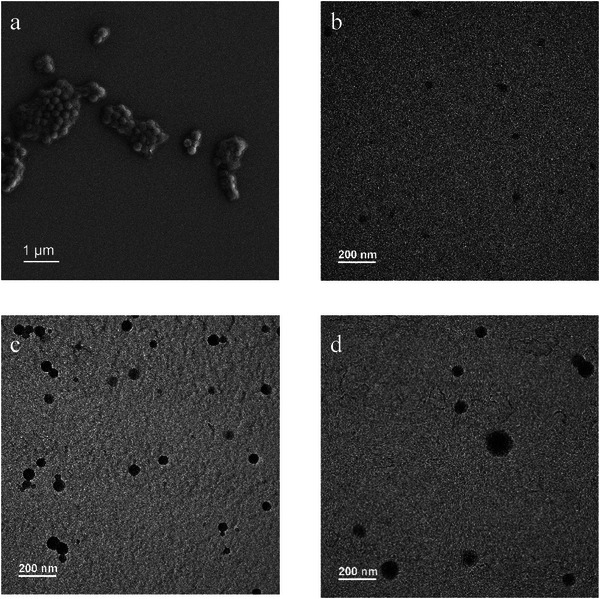
a) SEM image indicating the successful form of emulsion for the polymerization of isoprene in the presence of PMAA macroRAFT agent, b) TEM images of the PMAA‐*b*‐PI micelles, c) TEM image of PMAA‐*b*‐PS‐*b*‐PI micelles and d) PMAA‐*b*‐P(S‐*r*‐I) micelles. All the samples were directly taken from the emulsion.

PMAA‐*b*‐PI micelles could be well observed in the SEM image, as was shown in **Figure** **6**a and the TEM image of the sample in Figure 6b. Of course, due to the very low T_g_ of PMAA‐*b*‐PI, the micelles were very soft and not stable in TEM. The TEM images of PMAA‐*b*‐PS‐*b*‐PI and PMAA‐*b*‐P(S‐*r*‐I) are represented in Figure 6c and d, respectively. The presence of micelles serves as a significant indicator of successful emulsion polymerization. These micelles represent the self‐assembled structures formed during the polymerization process, where the hydrophobic polymer chains aggregate in an aqueous solution to form these distinct micelles. It is well‐known that the composition of the block copolymer, *i.e*., the ratio of the hydrophilic to the hydrophobic block, significantly controls the morphology of amphiphilic block copolymer nanoassemblies. Amphiphilic block copolymers carrying longer hydrophobic blocks form vesicles.^[^ ^60^, ^61^ ^]^ Therefore, our synthesized terpolymers with ≈13% hydrophilic block (PMAA) are more likely to adopt vesicle morphology. Due to the very low ratio of hydrophilic block, a distinct hydrophilic‐hydrophobic phase separation could not be observed within the images. Generally, the shape of the micelles further suggests a rather controlled and efficient polymerization process. The particle sizes according to the shown TEM images were found to be 45 ± 8, 55 ± 8 and 87 ± 25 for PMAA‐*b*‐PI, PMAA‐*b*‐PS‐*b*‐PI and PMAA‐*b*‐P(S‐*r*‐I), respectively. The particle size of all the samples was smaller in TEM images compared to DLS measurement, but still in good agreement and can be justified by the principal difference of the two methods. Overall, the presence of these micelles provides compelling evidence of the successful formation of polymer nanoparticles via emulsion polymerization.

## 3 Conclusion

PI homopolymer and also block and random copolymers with PS were synthesized using a PMAA macroRAFT agent, following the environmentally friendly synthesis technique of emulsion RAFT‐PISA polymerization in water. The polymerization conditions were optimized to achieve high conversion and molecular weight of the polymers, while also to avoid crosslinking reactions during the polymerization. Remarkably, through reducing the polymerization temperature to 35 °C, PMAA‐*b*‐PI (after 53 h), PMAA‐*b*‐PS‐*b*‐PI (after 24 h) and PMAA‐*b*‐P(S‐*r*‐I) (after 48 h) with a target molecular weight of 50 000 g mol^−1^ were synthesized with a monomer conversion of ≈80%. The dried polymers were soluble in THF, indicating the control over the crosslinking reaction that can occur during the polymerization process. According to ^1^H NMR, the microstructure of the polymers was mostly *1,4* for PI, with a PS/PI content of ≈45/55 and verified block and random copolymer structures. DSC analysis exhibited a distinct phase separation into high‐T*_g_* PS‐rich domains (106 °C) and low‐T*_g_* PI‐rich domains (‐57 °C) in PMAA‐*b*‐PS‐*b*‐PI while a single combined T*_g_* value at ‐0.6 °C in PMAA‐*b*‐P(S‐*r*‐I), confirming its random structure. Additionally, DLS and TEM demonstrated the successful emulsion polymerization, yielding nanoparticles with sizes ranging from 70 to 130 nm. The present study provides a reliable blue‐print to take advantage of the PISA technique for the synthesis and scale‐up of PI‐PS copolymers with different molecular weights and PS to PI ratio for the desired applications. Moreover, further improvement in polymerization rates and conversion could be achieved probably through, e.g., polymerization at higher pressures or the use of redox initiators at room temperature.

## 4 Experimental Section

### Materials

4,4′‐azobis(4‐cyanovaleric acid) (ACVA, ≥98%, Sigma–Aldrich, Germany, stored at 4 °C), 2,2′‐azobis[2‐(2‐imidazolin‐2‐yl)propane] dihydrochloride (VA044, >98.0%, TCI, Zwijndrecht, Belgium, stored at 4 °C), 4‐cyano‐4‐[(dodecylsulfanylthiocarbonyl)sulfanyl]pentanoic acid (CDTPA, 97%, abcr GmbH, Germany, stored at 4 °C), tetrahydrofuran‐*d*8 (THF‐*d*8, 99.5%, Deutero GmbH, Germany), diethyl ether (≥99.5%, Chemsolute, Renningen, Germany), THF (≥99.9%, Chemsolute, Renningen, Germany), *N,N*‐dimethyl formamide (DMF, 99.8%, Merck, Germany), ethanol (99%, VWR Chemicals, Germany), trifluoroacetic acid (TFA) (99%, TCI Chemicals, Eschborn, Germany), NaHCO_3_ (>99%, Grüssing, Filsum, Germany). Ultrapure MILLI‐Q water (σ > 18.2 MΩ cm^−1^) was obtained from a Millipore (Merck, Darmstadt, Germany) Direct‐Q UV water purification system. Methacrylic acid (MAA, ≥99%, stabilized with methoxyphenol, Sigma–Aldrich, Germany, stored at 4 °C) styrene (S, ≥99%, stabilized with 4‐tert‐butylcatechol, Sigma–Aldrich, Germany, stored at 4 °C), and isoprene (I, ≥99%, stabilized with 4‐tert‐butylcatechol, Sigma–Aldrich, Germany, stored at 4 °C) were freshly percolated through a column of basic aluminum oxide (activated, basic, Brockmann I, 100%, Honeywell, Seelze, Germany) to remove the inhibitor before use.

### Methods

*^1^H and ^13^C Nuclear Magnetic Resonance Spectroscopy*: ^1^H nuclear magnetic resonance (NMR) spectroscopy experiments were performed using a Bruker AV500 500 MHz spectrometer (Karlsruhe, Germany). ^1^H NMR spectra were recorded by applying a 30° pulse at a sample temperature of 298 K. A total of 16 scans were recorded with a relaxation delay of 5 s. The NMR spectra were analyzed with the software MestReNova 10.0 (Mestrelab Research, S.L., Santiago de Compostela, Spain).

The conversion of MAA was calculated from the decrease of the integrals of the vinyl peaks at 5.9 ppm (1 H) relative to the peak of DMF (internal reference) at 8.1 (1 H) in THF‐d8. The conversion of styrene in the emulsion polymerization, determined in THF‐d8, was calculated from the decrease of the vinyl peak at 5.2 ppm (1 H) relative to the integral of the phenylic hydrogens (from 6.9 to 7.5) as an internal reference.

^13^C NMR (45 dept in THF‐d8) was performed on the same instrumentation with a 256 total number of scans and a relaxation delay of 5 s at 298 K.

*Gel Permeation Chromatography (GPC)*: The apparent molar masses and molar mass distribution were obtained using GPC instrumentation at room temperature, using THF + 0.5 wt.% TFA as the eluent. A Waters 717 plus instrument equipped with Guard columns including column 1: particle size: 5 µm, mesh size: 100 Å, column 2: particle size: 5 µm, mesh size: 1000 Å and column 3: particle size: 5 µm, mesh size: 10 000 Å, was used. All the samples were prepared in THF with 0.5 wt.% TFA to ensure that the acid groups do not interact with the Guard columns. The samples were measured at a flow rate of 1 mL min^−1^ using a VWR‐Hitachi Primaide 1110 pump and a VWR‐Hitachi L‐2490 La Chrom Elite refractive index detector. GPC was calibrated with narrow PMMA (for PMAA) and PS standards (for PMAA‐*b*‐PS) from PSS GmbH (PSS GmbH, Mainz, Germany), and data were analyzed using the PSS WinGPC UniChrom (PSS GmbH, Mainz, Germany) software.

*Thermogravimetric analysis (TGA)*: TGA was conducted using a TG 209F1 Iris (NETZSCH GmbH, Selb, Germany) to observe the thermal stability of the synthesized polymers. A heating rate of 10 °C min^−1^ was used in a temperature interval of 25 to 600 °C. The derivate of the mass is defined as the differential thermogravimetry (DTG) and it is also included in the results.

*Differential scanning calorimetry (DSC)*: DSC 1 (Mettler Toledo, Gießen, Germany) calorimeter was used for DSC experiments and the software STARe SW 16.20 (Mettler Toledo, Gießen, Germany) was used for data evaluation. A 40 µL aluminum pan was filled with dried polymer and closed with a mono‐perforated lid. Three heating‐cooling‐heating cycles were carried out in a nitrogen atmosphere with a temperature interval from ‐70 to 200 °C under a nitrogen atmosphere. The heating/cooling rate for the cycles was 10 K min^−1^.

*Dynamic light scattering (DLS)*: The NANO‐flex II 180° DLS system (Meerbusch, Germany) was used to estimate the particle size of the emulsion polymerization. The dip‐in probe used in the NANO‐flex II had a diameter of 5.5 mm, and the principle of heterodyne 180° backscattering was applied to the calculation. The samples were directly withdrawn from the emulsions and diluted with MILLI‐Q water to obtain a final polymer concentration of 0.2% (w/w).

*Scanning electron microscopy (SEM)*: SEM images were obtained with the scanning electron microscope Merlin (ZEISS, Jena, Germany) at accelerating voltages of 0.7 to 2.0 kV. After polymerization, the samples were directly withdrawn from the emulsions and diluted with MILLI‐Q water to obtain a final polymer concentration of 0.2% (w/w) and then drop‐cast on silicon wafer. Before measurement the samples were sputtered with a few nanometers of platinum coating to prevent sample charging.

*Transmission electron microscopy*: TEM images of the polymer particles were recorded with a Tecnai G2 F20 electron microscope (Thermo Fisher Scientific, Amsterdam, The Netherlands) at an accelerating voltage of 120 kV. The samples were directly withdrawn from the emulsions after polymerization and diluted with MILLI‐Q water to obtain a final polymer concentration of 0.2% (w/w). 2 µL of the diluted dispersions were drop‐cast onto carbon‐film‐coated TEM grids (Plano, Wetzlar, Germany).

### Synthesis of PMAA macroRAFT Agent by RAFT Solution Polymerization

A typical synthesis of PMAA (with target *M̅*_n_ of 8500) via RAFT solution polymerization was conducted according to the literature^[^ ^38^ ^]^ as follows: CDTPA (0.26 g, 0.64 mmol, 9.5 eq.) and ACVA (0.019 g, 0.068 mmol, 1 eq.) were dissolved in ethanol (22.5 g, 30 mmol). Following that, 5.304 g (61.6 mmol, 909 eq.) MAA was added to the mixture ([MAA]/[CDTPA]/[ACVA] = 909/9.5/1) with 0.3 ml DMF that was used as the internal standard for conversion calculation. The solution was degassed by purging with nitrogen for 15 min at 0 °C. The polymerization reaction was conducted in a thermoshaker (CellMedia, Germany) at 80 °C and 850 rpm for 9 h and then quenched by ice‐cooling and exposure to air. Subsequently, the reaction solution was precipitated in ice‐cold diethyl ether. After that, the polymer was dried, dissolved in THF, and precipitated in diethyl ether. Afterwards, the polymer was dried in vacuum at 30 °C for 24 h and obtained as a light‐yellow powder. A final PMAA conversion of 77% was calculated using ^1^H NMR spectroscopy. The corresponding ^1^H NMR spectrum of the final product was shown in Figure S1 (Supporting Information). The number average molecular weight was determined via ^1^H NMR and conversion: *M̅*_n_^NMR^ = 6600 g mol^−1^. Apparent average molecular weight obtained by GPC: *M̅*_n_^GPC^ = 5500 g mol^−1^) and molecular weight dispersity: *Đ* = 1.1 (PMMA standard).

### Synthesis of PMAA‐*b*‐PI (50 000 g mol^−1^) via RAFT Emulsion Polymerization

For the synthesis of PMAA‐*b*‐PI first VA044 (0.00245 g, 0.0076 mmol, 1 eq) was dissolved in water (6.4 g). Then PMAA macroRAFT agent/macrostabilizer (0.265 g, 0.040 mmol, 5.3 eq) was added to the solution and the solution was transferred to the polymerization ampul. Afterwards, isoprene (1.83 g, 27 mmol, 3545 eq) was added to the solution in the ampul (25 wt.% solid content). Due to the low boiling point of isoprene (39 °C) the polymerization was conducted using three freeze‐pump‐thaw technique within an evacuated ampul to prevent isoprene evaporation which can occur during both nitrogen gas bubbling for oxygen removal and polymerization in a polymerization glass vial. The polymerization was carried out through stirring at 800 rpm using a thermoshaker at 35 °C for 53 h. Notably, the polymerization solution had two individual phases which turned to a one phase milky solution when reaching to high conversions (Figure S2, Supporting Information), therefore, due to the two‐phase polymerization solution ^1^H NMR sample could not be taken in the intervals for the conversion analysis. The polymerization was quenched by ice‐cooling and exposure to air. The crude polymer was isolated by removing water under reduced pressure. ^1^H NMR and ^13^C NMR of the dried polymer were shown in Figure S5 and S6 (Supporting Information), respectively. ^13^C NMR was evaluated according to the literature ad the chemical shifts were presented in Table S1 which.^[^ ^39^ ^]^ Based on the product weight, the yield of the polymerization was 84%. *M̅*_n_^theo^ = 43 000 g mol^−1^, *M̅*_n_^GPC^ = 70 000 g mol^−1^ (PI standard), *Đ* = 1.7.

### Synthesis of PMAA‐*b*‐P(S‐*r*‐I) (50 000 g mol^−1^) via RAFT Emulsion Polymerization

For the synthesis of PMAA‐*b*‐P(S‐*r*‐I) the same procedure as PMMA‐*b*‐PI synthesis was followed. In a polymerization ampul VA044 (0.0033 g, 0.0102 mmol, 1 eq) was dissolved in water (7.9 g). Then PMAA macroRAFT agent/macrostabilizer (0.3360 g, 0.051 mmol, 5 eq) was added to the solution. Afterwards, styrene (1.2 g, 11 mmol, 1130 eq) and isoprene (1.3 g, 19 mmol, 1870 eq) were added followed by freeze‐pump‐thaw degassing. The target feed ratio of S/I was 0.4, but a small amount of additional isoprene was added to compensate the probable evaporation of isoprene (solid content: 20 wt.%). Polymerization continued for 48 h at 35 °C. ^1^H NMR of the dried polymer was shown in Figure 3a. ^13^C NMR of the dried polymer was shown in Figure S7 and the chemical shifts were peresented inTable S2 (Supporting Information) and was evaluated according to the literature.^[^ ^39^ ^]^ Based on the product weight evaluation, the yield of the polymerization was 82%. Rough *M̅*_n_^theo^ = 42 000 g mol^−1^, *M̅*_n_^GPC^ = 53 700 g mol^−1^ (PS standard), *Đ* = 2.4

### Synthesis of PMAA‐*b*‐PS‐*b*‐PI (50 000 g mol^−1^) via RAFT Emulsion Polymerization

PMAA‐*b*‐PS‐*b*‐PI was synthesized in two steps. First, PMAA‐*b*‐PS was prepared through emulsion polymerization following the literature,^[^ ^38^ ^]^ which was subsequently extended with PI block. For the synthesis of PMAA‐*b*‐PS with a target molecular weight of 23 200 g mol^−1^, ACVA (0.0026 g, 0.00928 mmol, 1 eq) together with additional amount of NaHCO_3_ (0.0027 g, 0.0325 mmol) (for the dissolution of ACVA in water) were dissolved in water (4.3 g) in a polymerization vial. Then PMAA macroRAFT agent/macrostabilizer (0.3000 g, 0.0456 mmol, 5 eq) was dissolved in the solution and then styrene (0.800 g, 7.40 mmol, 861 eq) was added to the solution (solid content: 20 wt.%). The solution was degassed by purging with nitrogen for 15 min at 0 °C. The polymerization reaction was conducted in a thermoshaker at 75 °C and 800 rpm for 2.30 h with a full conversion, using ^1^H NMR spectroscopy, and then quenched by ice‐cooling and exposure to air. The corresponding ^1^H NMR spectrum of the final product is shown in Figure S8 (Supporting Information). *M̅*_n_^NMR^ = 24 100 g mol^−1^, *M̅*_n_^GPC^ = 31 000 g mol^−1^, and *Đ* = 1.5 (PS standard).

Afterwards, the synthesized PMAA‐*b*‐PS emulsion was directly used for the chain extension with PI block, reaching to the theoretical molecular weight of 50 000 g mol^−1^ and PS/PI ratio of 0.4. To this end, 4.7 g of PMAA‐*b*‐PS emulsion which contains 0.94 g, 0.0371 mmol (10 eq) PMAA‐*b*‐PS was transferred to the polymerization ampul. Then VA044 (0.0012 g, 0.0037 mmol, 1 eq) was dissolved in 3.67 g water, considering 3.76 g water in PMAA‐*b*‐PS emulsion which results in 7.43 g water for the final solid content of 20 wt.%. Then, isoprene (1.1 g, 13.3 mmol, 4350 eq) was added to the solution in the ampule followed by the same procedure as PMAA‐*b*‐PI synthesis. Polymerization continued for 24 h at 35 °C. ^1^H NMR of the dried polymer was shown in Figure 3b. ^13^C NMR of the dried polymer shown in Figure S9 and the chemical shifts were presented in Table S3 (Supporting Information). Based on the product weight, the yield of the polymerization was 73%. *M̅*_n_^theo^ = 44 500 g mol^−1^, *M̅*_n_^GPC^ = 49 000 g mol^−1^ (PI standard), *Đ* = 2.1.

## Conflict of Interest

The authors declare no conflict of interest.

## Supporting information



Supporting Information

## Data Availability

The data that support the findings of this study are available from the corresponding author upon reasonable request.
