# Supplementary material for: Polymerization‐Induced Self‐Assembly for the Synthesis of Polyisoprene‐Polystyrene Block and Random Copolymers: Towards High Molecular Weight and Conversion
Source: Macromol Rapid Commun. 2024 Oct 26;46(3):2400727. doi: 10.1002/marc.202400727 (PMC11800055; doi:10.1002/marc.202400727)
Supplement: Supplementary file 1 — Supporting Information [file MARC-46-2400727-s001.docx]

Supporting Information

Polymerization-Induced Self-Assembly for the Synthesis of Polyisoprene-Polystyrene Block and Random Copolymers: Towards High Molecular Weight and Conversion

*Maryam Moradi, Prokopios Georgopanos**

M. Moradi, P. Georgopanos

Helmholtz-Zentrum Hereon

Institute of Membrane Research

Max-Planck-Straße 1, 21502 Geesthacht, Germany
E-mail: [prokopios.georgopanos@hereon.de](mailto:prokopios.georgopanos@hereon.de)

**Figure S1.** ^1^H-NMR spectra (500 MHz, THF-d8) of PMAA macroRAFT.

1. b)


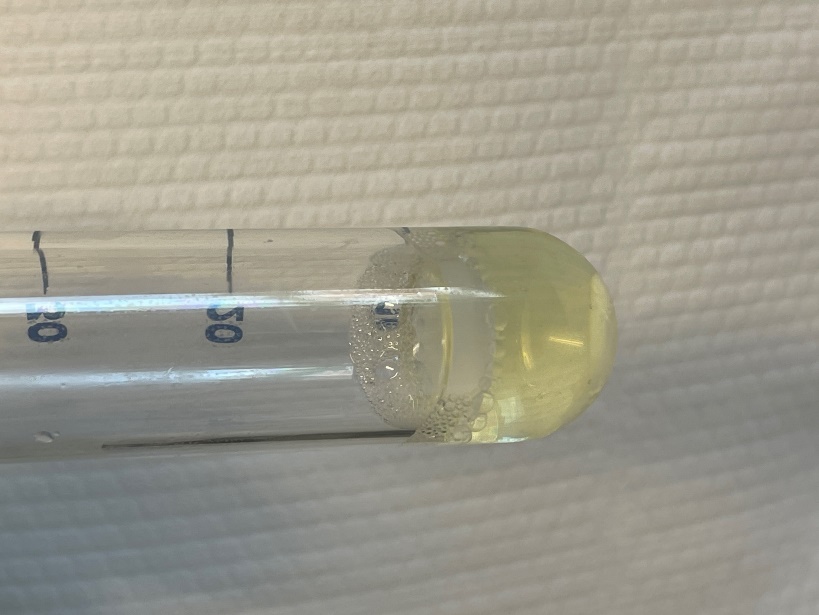

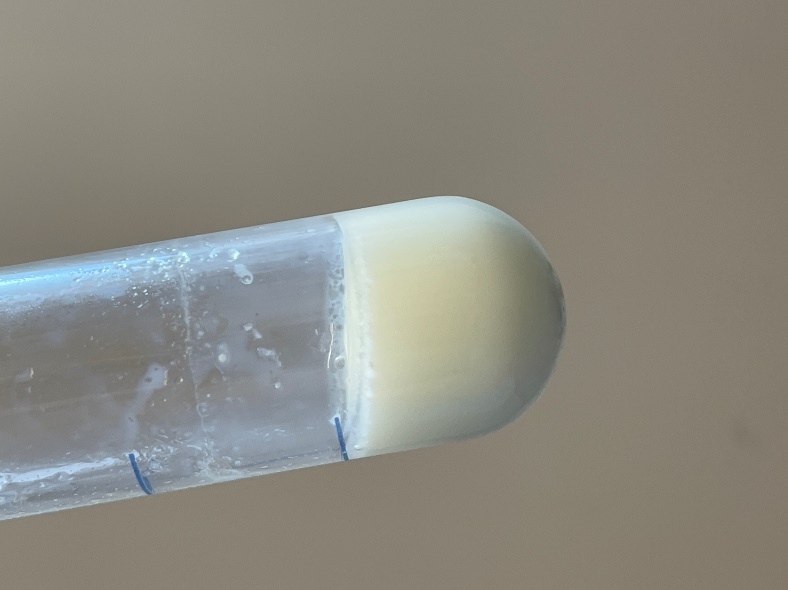


Isoprene

PMAA macroRAFT solution in water

**Figure S2.** Images of the polymerization solutions of PMAA-b-PI a) before and b) after polymerization.


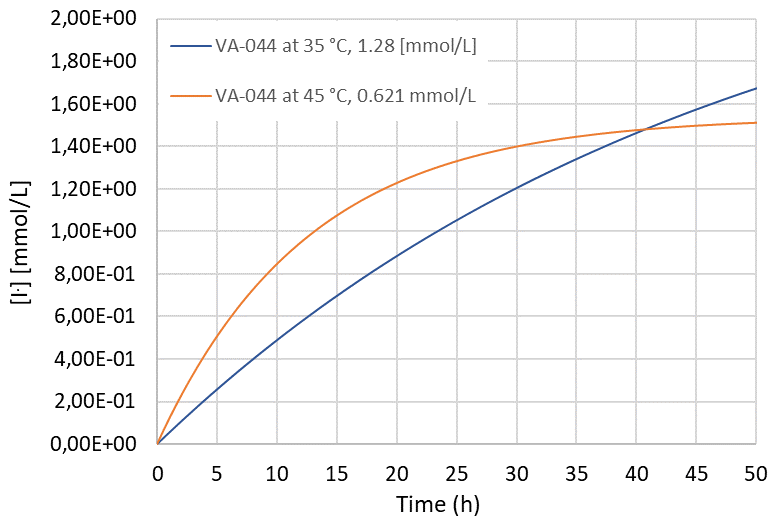


**Figure S3.** Comparison of radical concentration of VA044 at 35 °C and 45 °C using decomposition constant rate of 5.88 E-06 at 35 °C and 2.22 E-05 at 45 °C and initial concentration of VA044: 1.28 mmol L^-1^ at 35 °C and 0.621 mmol L^-1^ at 45 °C. After 41 hours, the radical concentration reaches equilibrium at both temperatures.


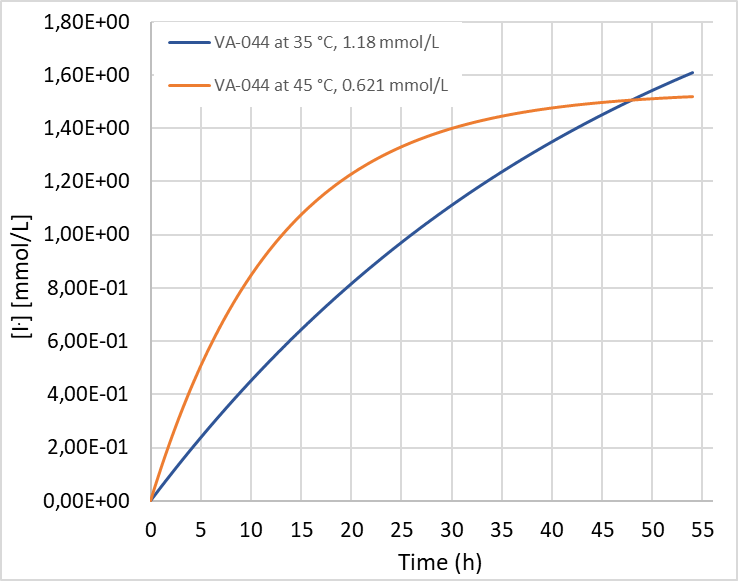


**Figure S4**. Comparison of radical concentration of VA044 at 35 °C and 45 °C using decomposition constant rate of 5.88 E-06 at 35 °C and 2.22 E-05 at 45 °C and initial concentration of VA044: 1.18 mmol L^-1^ at 35 °C and 0.621 mmol L^-1^ at 45 °C. After 48 hours, the radical concentration reaches equilibrium at both temperatures.

**Figure S5.** ^1^H NMR spectra (500 MHz, THF-d8) of PMAA-b-PI.

**Evaluation of homo-polymers composition**

**PMAA-*b*-PI ( sample 8)**

Polyisoprene composition was evaluated according to the areas under the corresponding hydrogen peak of each microstructure as below:

A = area under the peaks.

1,4 = (A_1,4_) / (A_1,4_ + A_1,2_/2 + A_3,4_/2) × 100 1 / (1 + 0.06 + 0.06) × 100 = 89%

1,2 = (A_1,2_/2) / (A_1,4_ + A_1,2_/2 + A_3,4_/2) × 100 0.06 / (1 + 0.06 + 0.06) × 100 = 5.5%

3,4 = (A_3,4_/2) / (A_1,4_ + A_1,2_/2 + A_3,4_/2) × 100 0.06 / (1 + 0.06 + 0.06) × 100 = 5.5%

**PMAA-*b*-P(S-*r*-I)**

Polyisoprene composition was evaluated according to the areas under the corresponding hydrogen peak of each microstructure as below:

S% = (A_S_/5) / (A_S_/5 + A_1,4_ + (A_1,2_ + A_3,4_)/2) × 100 0.20 / (0.20 + 0.218 + 0.030) × 100 = 45% I%=100-45=55%

A = area under the peaks.

1,4 = (A_1,4_) / (A_1,4_ + (A_1,2_ + A_3,4)_/2) × 100 0.218 / (0.218 + 0.03) × 100 = 88%

1,2 + 3,4 = (A_1,2_ + A_3,4_)/2 / (A_1,4_ + (A_1,2_ + A_3,4_)/2) × 100 0.03 / (0.218 + 0.03) × 100 = 12%

**PMAA-*b*-PS-*b*-PI**

Polyisoprene composition was evaluated according to the areas under the corresponding hydrogen peak of each microstructure as below:

S% = (A_S_/5) / (A_S_/5 + A_1,4_ + A_1,2_/2 + A_3,4_/2) × 100 0.20 / (0.20 + 0.201 + 0.013 + 0.013) × 100 = 47% I%=100-47=53%

A = area under the peaks.

1,4 = (A_1,4_) / (A_1,4_ + A_1,2_/2 + A_3,4_/2) × 100 0.201 / (0.201 + 0.013 + 0.013) × 100 = 88%

1,2 = (A_1,2_/2) / (A_1,4_ + A_1,2_/2 + A_3,4_/2) × 100 0.013 / (0.2 + 0.013 + 0.013) × 100 = 6%

3,4 = (A_3,4_/2) / (A_1,4_ + A_1,2_/2 + A_3,4_/2) × 100 0.013 / (0.2 + 0.013 + 0.013) × 100 = 6%

The evaluation of ^13^C-NMR spectra was done according to the previous publication as mentioned in the experimental part.

**Figure S6.** ^13^C-NMR spectra (500 MHz, THF-d8) of PMAA-b-PI.

**Table S1.** ^13^C-NMR chemical shift of PMAA-*b*-PI.

| **Structure unit** | **Carbon** | **Chemical Shift** |
| --- | --- | --- |
|  | T-**T1**-T | 39.71 |
|  | V-**T1**-T/C | 38.2 |
|  | C-**T1**-T/C | 39.74 |
|  | T/C-**C1**-T/C | 31.82 |
|  | T/C-**V1**-T/C | 110.94 |
|  | T/C-**T2**-T/C | 135.01 |
|  | T/C-**C2**-T/C | 135.01 |
|  | T/C-**V2**-T/C | 147.67 |
|  | T/C-**T3**-T/C | 124.18 |
|  | T/C-**C3**-T/C | 124.93 |
|  | T/C-**V3**-T/C | 44.58 |
|  | T/C-**T4**-T/C | 26.62 |
|  | V-**T4**-T/C | 32.67 |
|  | V-**C4**-T/C | 30.77 |
|  | C/T-**V4**-T/C | 28.18 |
|  | T/C-**T5**-T/C | 15.22 |
|  | T/C-**C5**-T/C | 22.77 |
|  | T/C-**V5**-T/C | 18.13 |

**Figure S7.** ^13^C-NMR spectra (500 MHz, THF-d8) of PMAA-b-P(S-r-I).

**Table S2.** ^13^C-NMR chemical shift of PMAA-*b*-P(S-*r*-I)

| **Structure unit** | **Carbon** | **Chemical Shift** |
| --- | --- | --- |
|  | S**-S1**-S | 43.96–47.86 |
|  | T/C**-S1**-T/C | 35.85 |
|  | T/C**-S1**-S | 43.74 |
|  | V**-S1**-V | 48.47 |
|  | S**-T1**-S/T/C | 38.50 |
|  | T**-T1**-T | 39.73 |
|  | T**-T1**-C | 40.49 |
|  | S-**C1**-S | 29.76 |
|  | T/C-**C1**-T | 31.87 |
|  | T/C/S-**V1**-T/C/S | 110.99 |
|  | S**-S2**-S | 40.49 |
|  | C/T**-S2**-C/T | 42.27-43.97 |
|  | S/T/C-**V2**-S/T/C | 147.69 |
|  | C/T/S**-T3/C3**-C/T/S | 124.19 |
|  | T/C-**V3**-T/C | 47.69 |
|  | S**-V3**-S | 48.47 |
|  | C/T/S**-S4/S5**-C/T/S | 127.17-128.85 |
|  | C/T/S**-T4**-S | 25.80 |
|  | T**-T4**-T/C | 26.64 |
|  | T/C-**V4**-T/C | 28.17 |
|  | S/T/C-**T5**-S/T/C | 15.29 |
|  | S/T/C-**C5**-S/T/C | 22.80 |
|  | S/T/C-**V5**-S/T/C | 18.13 |
|  | S/T/C-**S6**-S/T/C | 125.67 |

**Figure S8.** ^1^H-NMR spectra (500 MHz, THF-d8) of PMAA-b-PS.

**Figure S9**. ^13^C-NMR spectra (500 MHz, THF-d8) of PMAA-b-PS-b-PI.

**Table S3.** ^13^C-NMR chemical shift of PMAA-*b*-PS-*b*-PI

| **Structure unit** | **Carbon** | **Chemical Shift** |
| --- | --- | --- |
|  | S**-S1**-S | 43.80–47.00 |
|  | T/C**-S1**-S | 43.96 |
|  | V**-S1**-V | 46.60 |
|  | S**-T1**-S/T/C | 38.50 |
|  | T**-T1**-T | 39.71 |
|  | T**-T1**-C | 40.43 |
|  | S-**C1**-S | 29.68 |
|  | T/C-**C1**-T | 31.84 |
|  | T/C/S-**V1**-T/C/S | 110.67 |
|  | S**-S2**-S | 40.43 |
|  | C/T**-S2**-C/T | 42.00-43.58 |
|  | S/T/C-**V2**-S/T/C | 147.69 |
|  | C/T/S**-T3/C3**-C/T/S | 124.19 |
|  | T/C-**V3**-T/C | 46.60 |
|  | S**-V3**-S | 46.10 |
|  | C/T/S**-S4/S5**-C/T/S | 127.0-128.8 |
|  | C/T/S**-T4**-S | 25.77 |
|  | T**-T4**-T/C | 26.64 |
|  | T/C-**V4**-T/C | 28.17 |
|  | S/T/C-**T5**-S/T/C | 15.22 |
|  | S/T/C-**C5**-S/T/C | 22.77 |
|  | S/T/C-**V5**-S/T/C | 18.13 |
|  | S/T/C-**S6**-S/T/C | 125.46 |
